# Urinary Metabolite Profiling Offers Potential for Differentiation of Liver-Kidney Yin Deficiency and Dampness-Heat Internal Smoldering Syndromes in Posthepatitis B Cirrhosis Patients

**DOI:** 10.1155/2015/464969

**Published:** 2015-01-18

**Authors:** Xiaoning Wang, Guoxiang Xie, Xiaoyan Wang, Mingmei Zhou, Huan Yu, Yan Lin, Guangli Du, Guoan Luo, Ping Liu

**Affiliations:** ^1^E-Institute of Shanghai Municipal Education Committee, Shanghai University of Traditional Chinese Medicine, Shanghai 201203, China; ^2^Key Laboratory of Liver and Kidney Diseases, Ministry of Education, Institute of Liver Diseases, Shuguang Hospital, Shanghai University of Traditional Chinese Medicine, Shanghai 201203, China; ^3^University of Hawaii Cancer Center, Honolulu, HI 96813, USA; ^4^Key Laboratory of Systems Biomedicine, Ministry of Education, Shanghai Center for Systems Biomedicine, Shanghai Jiao Tong University, Shanghai 200240, China; ^5^Center for Chinese Medical Therapy and Systems Biology, Shanghai University of Traditional Chinese Medicine, Shanghai 201203, China; ^6^Key Laboratory of Bioorganic Phosphorus Chemistry and Chemical Biology, Ministry of Education, Department of Chemistry, Tsinghua University, Beijing 100083, China; ^7^School of Traditional Chinese Medicine, Beijing University of Chinese Medicine, Beijing 100102, China

## Abstract

Zheng is the basic theory and essence of traditional Chinese medicine (TCM) in diagnosing diseases. However, there are no biological evidences to support TCM Zheng differentiation. In this study we elucidated the biological alteration of cirrhosis with TCM “Liver-Kidney Yin Deficiency (YX)” or “Dampness-Heat Internal Smoldering (SR)” Zheng and the potential of urine metabonomics in TCM Zheng differentiation. Differential metabolites contributing to the intergroup variation between healthy controls and liver cirrhosis patients were investigated, respectively, and mainly participated in energy metabolism, gut microbiota metabolism, oxidative stress, and bile acid metabolism. Three metabolites, aconitate, citrate, and 2-pentendioate, altered significantly in YX Zheng only, representing the abnormal energy metabolism. Contrarily, hippurate and 4-pyridinecarboxylate altered significantly in SR Zheng only, representing the abnormalities of gut microbiota metabolism. Moreover, there were significant differences between two TCM Zhengs in three metabolites, glycoursodeoxycholate, cortolone-3-glucuronide, and L-aspartyl-4-phosphate, among all differential metabolites. Metabonomic profiling, as a powerful approach, provides support to the understanding of biological mechanisms of TCM Zheng stratification. The altered urinary metabolites constitute a panel of reliable biological evidence for TCM Zheng differentiation in patients with posthepatitis B cirrhosis and may be used for the potential biomarkers of TCM Zheng stratification.

## 1. Introduction

Cirrhosis is scarring of the liver and also is the final stage of many chronic liver diseases, leading to portal hypertension and end-stage liver disease [1]. Over the past decades, a series of methods, including serum biochemical tests [2], abdominal imaging scan [3], and liver biopsy [4], have been developed for the diagnosis of liver cirrhosis. To date, liver biopsy is still considered to be a golden standard for diagnosing cirrhosis, although it is a high-risk invasive surgery [5–8]. In clinic, imaging scan such as computed tomography (CT) and ultrasound or transient elastography are noninvasive methods, getting more and more attention due to the excellent diagnostic accuracy in assessing the pathological stage of liver fibrosis [9–11]. Although noninvasive, none of them can provide the information on inflammatory activity, steatosis, or other findings derived from liver biopsy [12]. Child-Pugh (CP) scores classification is a widespread method to grade the liver function levels in cirrhotic patients [13, 14]. It can reasonably predict survival in many chronic liver conditions and the likelihood of major complications such as bleeding from varices and spontaneous bacterial peritonitis and is still considered to be a cornerstone in prognostic evaluation of cirrhosis [15, 16]. However, the CP score does not provide direct evidence of the pathological stage or state of cirrhosis [2]. Moreover, it has some drawbacks such as the limited discriminatory ability as well as the fact that it depends greatly on the clinician's experience [3, 4].

Zheng (Chinese character transliteration) is a temporary state at one time and is also known as a traditional Chinese medicine (TCM) syndrome [17]. It is, in essence, a characteristic profile of all clinical manifestations, such as signs, symptoms, and all other presentative information, even psychology, emotion, feeling, and so forth that can be identified by a TCM practitioner. Syndrome differentiation (TCM Zheng) is an important element in TCM theories and is the basis for the treatments of all diseases, including cirrhosis. In TCM, patients with posthepatitis B cirrhosis will usually be classified into different Zhengs, “Liver-Kidney Yin Deficiency,” “Dampness-Heat Internal Smoldering,” “Stasis-Heat Internal Smoldering,” “Liver Depression and Spleen Deficiency,” and “Spleen-Kidney Qi Deficiency” [18–21], by clustering and merging the uniform or analogical clinical evidences. Importantly, clinical investigations reveal that the total effective rate is significantly higher in those subject to integrated traditional Chinese and western medicine treatment based on TCM Zheng stratification than those only subject to western medicine [22–24]. Since TCM Zheng stratification significantly improves the clinical therapeutic outcome of liver cirrhosis, the TCM syndromes (Zheng) of patients with cirrhosis are necessary to characterize [25]. However, in clinic, TCM Zheng identification commonly relied on the experience of TCM practitioners. Recent advance in systems biology provides a panel of platform for the joint development of multidisciplinary partnerships. Metabonomics, a quantitative determination of the multiparametric metabolic response of living systems [26, 27], has been widely accepted as an effective approach for the study of pathophysiological changes associated with or resulting from disease or injury [28]. It can provide abundant mechanistic information to achieve the diagnosis of disease and the curative effect evaluation of drugs [29–31] by a variety of endogenous substances differentially expressed in tissues or biofluids such as blood and urine [32–34]. In our recent study [35], metabonomics approach showed that urinary metabolite variation is closely associated with pathological progression of liver cirrhosis in posthepatitis B cirrhosis patients.

In this study, we conduct a urinary metabonomic study on a group of liver cirrhosis patients (*n* = 63) and healthy subjects (*n* = 31), the same participants used in our previous study [35], using a combined gas chromatography-mass spectrometry (GC-MS) and ultraperformance liquid chromatography quadrupole time-of-flight mass spectrometry (UPLC-QTOFMS). Our study is aimed at (1) comparing the urinary metabolic profiles of participants with and without cirrhosis, (2) illustrating the relationship between urine metabolic profiles and TCM syndromes in subjects with cirrhosis, and (3) determining the characteristics and differences in TCM syndrome distribution between Liver-Kidney Yin Deficiency and Dampness-Heat Internal Smoldering.

## 2. Materials and Methods

### 2.1. Participants

We used a multicenter, multistage sampling method to obtain a cohort of representative samples of male patients in the general liver cirrhosis population. Patients were eligible to enter the study if they were clinically diagnosed liver cirrhosis due to chronic hepatitis B infection according to the “Guideline on prevention and treatment of chronic hepatitis B in China (2005).” The guideline (2005 version) was jointly revised in 2007 by Chinese Society of Hepatology, Chinese Medical Association, and Chinese Society of Infectious Diseases, Chinese Medical Association [36]. In addition, patients must match the factors of TCM Zheng diagnosis criteria of posthepatitis B cirrhosis [18–20], in which “Liver-Kidney Yin Deficiency” Zheng (*shorter form: YX*) included blurred vision, tinnitus, xerophthalmia, skin itching, bitter taste and dry mouth, poor libido, soreness and flaccidity of waist and knees, constipation, rapid pulse and vexing heat in the chest, palms, and soles, while “Dampness-Heat Internal Smoldering” Zheng (*shorter form: SR*) included yellow and slimy tongue fur, jaundice in the skin and sclera, yellow urine, abdominal distension, edema of lower limbs, gynecomastia, dim facial complexion, fatigue, and heavy body.

Exclusion criteria in the study were patients having a history of hepatitis A, C infection, alcohol or drug abuse, liver cancer, neoplastic liver diseases, hepatotoxic medication, and autoimmune liver disease in the past 6 months before recruiting into the study and other conditions likely to interfere with the study, such as overt hepatic encephalopathy (West Heaven Criteria grade II through IV), spontaneous bacterial peritonitis, upper gastrointestinal haemorrhage, and hepatorenal syndrome. Those with a history of severe primary heart, brain, lung, spleen, kidney, endocrine diseases, hematological disorder, and psychosis were also excluded from the study. Meanwhile, those not matching TCM YX Zheng or SR Zheng diagnosis criteria of cirrhosis were ruled out.

A total of 63 patients, aged between 33 and 58, were enrolled in the study from Shuguang Hospital, Longhua Hospital, and Putuo District Center Hospital affiliated to Shanghai University of Traditional Chinese Medicine (Shanghai, China) and Shanghai Public Health Center affiliated to Fudan University (Shanghai, China) between January 1, 2007, and December 31, 2008. All patients were clinically stable at the time of assessment. Patients were spontaneously divided into YX Zheng subgroup (*n* = 33) and SR Zheng subgroup (*n* = 30), according to TCM diagnosis criteria of cirrhosis.

A cohort of 31 male participants was recruited as healthy controls from the Physical Examination Center of Shuguang Hospital. There was no significant difference in age, height, body weight, and BMI between healthy controls and liver cirrhosis patients or between TCM YX Zheng and SR Zheng of cirrhosis patients (Table 1). The injury degrees of liver function in cirrhosis patients with TCM YX and SR Zheng were approximately equal according to Child-Pugh liver function classification.

Ethical approval for these studies was obtained from the ethics committees of the four hospitals mentioned above. The study was carried out in compliance with the Declaration of Helsinki (55th World Medical Association General Assembly, Tokyo, 2004). All participants have written the informed consent prior to the study.

### 2.2. Study Design

Before the study, all study investigators, including medical students, trained general practitioners, and nurses, had completed a training program for methods and requirements of samples collection and obtained a manual of detailed procedure that guided how to manage the questionnaires, anthropometric measurements, and biological samples (urine and serum). All participants completed a questionnaire documenting their anthropometric measurements (e.g., weight and height), sociodemographic status (e.g., age, sex, education, and career), personal and family health history (e.g., hypertension, diabetes, liver disease, and surgery), lifestyle (e.g., smoking and alcohol consumption), and TCM Zheng scale (published in http://www.hindawi.com/journals/ecam/2012/496575/) under the guidance of the study investigators. Other information in the inclusion and exclusion criteria was noted. Serum samples of all patients were obtained under the fasting status in the morning. Clean voided midstream urine samples were obtained in the morning before breakfast and stored at −80°C condition in Key Laboratory of Liver and Kidney Diseases (Ministry of Education), until GC-MS or UPLC-QTOFMS analysis.

Serum biochemical assay was performed with an automatic biochemistry analyzer for the analysis of blood routine, liver, and renal function markers. The data were offered by clinical laboratory of each hospital participating in the study. All questionnaires and serum biochemical indices were stored and analyzed by Key Laboratory of Liver and Kidney Diseases (Ministry of Education), Institute of Liver Diseases, Shuguang Hospital, Shanghai, China.

GC-MS profiling and data analysis of urine samples completed by Center for Chinese Medical Therapy and Systems Biology, Shanghai University of Traditional Chinese Medicine, Shanghai, China. The urine sample preparation for GC-MS analysis was performed according to our previously published method with minor modification [37]. L-2-chlorophenylalanine was used as internal standard (IS) to monitor GC/MS performance and method reproducibility during a long time of run.

Raw GC-MS data were converted into AIA format (NetCDF) files by Agilent GC-MS 5975 Data Analysis software, and subsequently the data information was extracted by the XCMS toolbox using the parameters as previously described. The XCMS output (TSV file) was introduced to Matlab software version 7.0 (The MathWorks, Inc.), where internal standard (IS) peaks and impurity peaks from column bleeds and derivatization procedure were excluded. The remaining ion features with high correlation of abundance within the same retention time group were combined into a single compound so as to obtain the total numbers of compounds and simplify data matrix for multivariate statistical analysis. The intensities of ion features (area) were further normalized to the total area for each sample to eliminate the variations caused by the different volume of individual urine sample and arranged on a three-dimensional matrix consisting of arbitrary peak index (RT-*m*/*z* pair), sample names (observations), and peak area (variables).

UPLC-QTOFMS profiling and data analysis of urine samples were completed by Key Laboratory of Bioorganic Phosphorus Chemistry and Chemical Biology (Ministry of Education), Department of Chemistry, Tsinghua University, Beijing, China. The urine sample preparation for UPLC-QTOFMS was performed according to our previous works [38]. A 100 *μ*L of each urinary sample was mixed with 400 *μ*L of methanol and vortexed for 2 min followed by centrifugation at 6,000 rpm for 15 min at 4°C. The clear supernatant was transferred to a separate container and diluted with ultrapure water (1 : 3) before analysis.

A Waters ACQUITY UPLC system coupled with an orthogonal acceleration time-of-flight mass spectrometry equipped with an electrospray interface (Waters Corp., Milford, USA) was used for metabonomic profiling. Chromatographic separation was performed on a Waters ACQUITY BEH C18 column (100 × 2.1 mm, 1.7 *μ*m) maintained at 50°C with a mobile phase consisting of (A) 0.1% formic acid in water and (B) acetonitrile. A linear gradient was set as follows: 0–3 min, 5% B to 50% B; 3–20 min, 50% B to 95% B; 20-21 min, 95% B to 5% B; 21–23 min, equilibration with 5% B with a flow rate of 0.4 mL/min. Mass spectrometry conditions were as follows: negative ion electrospray ionization (ESI-) mode, capillary voltage 2.5 kV, sample cone voltage 50 V, desolvation temperature 350°C, source temperature 120°C, cone gas flow 40 L/h, desolvation gas flow 700 L/h, and MCP detector voltage 2.2 kV. The data acquisition rate was set to 0.1 s, with a 0.05 s interscan delay using dynamic range enhancement (DRE). All analyses were acquired using the lock spray to ensure accuracy and reproducibility; leucine-enkephalin was used as the lock mass (*m*/*z* 554.2615) at a concentration of 50 pg/mL and an infusion flow rate of 5 *μ*L/min. Data was collected in centroid mode from 50 to 1000 *m*/*z*.

The peak picking, peak alignment, and peak filtering of the raw UPLC-QTOFMS data were carried out with the MarkerLynx Application Manager Version 4.1 (Waters, Manchester, UK). The parameters used were retention time range 0–18 min, mass range 50–1000 *m*/*z*, mass tolerance 0.05 Da, intensity threshold 10 counts, and retention time tolerance 0.01 min; noise elimination level was set at 6.00 and isotopic peaks were excluded for processing.

Compound annotation from UPLC-QTOFMS data was performed by comparing the accurate mass (*m*/*z*) and retention time (Rt) of reference standards in our in-house library and the accurate mass of compounds obtained from the web-based resources such as the Human Metabolome Database (http://www.hmdb.ca/). For GC-TOFMS data, compound annotation was carried out by comparing the mass fragments and Rt with our in-house library or mass fragments with NIST 05 Standard mass spectral databases in NIST MS search 2.0 (NIST, Gaithersburg, MD) software with a similarity of greater than 70%.

**Figure 1:**
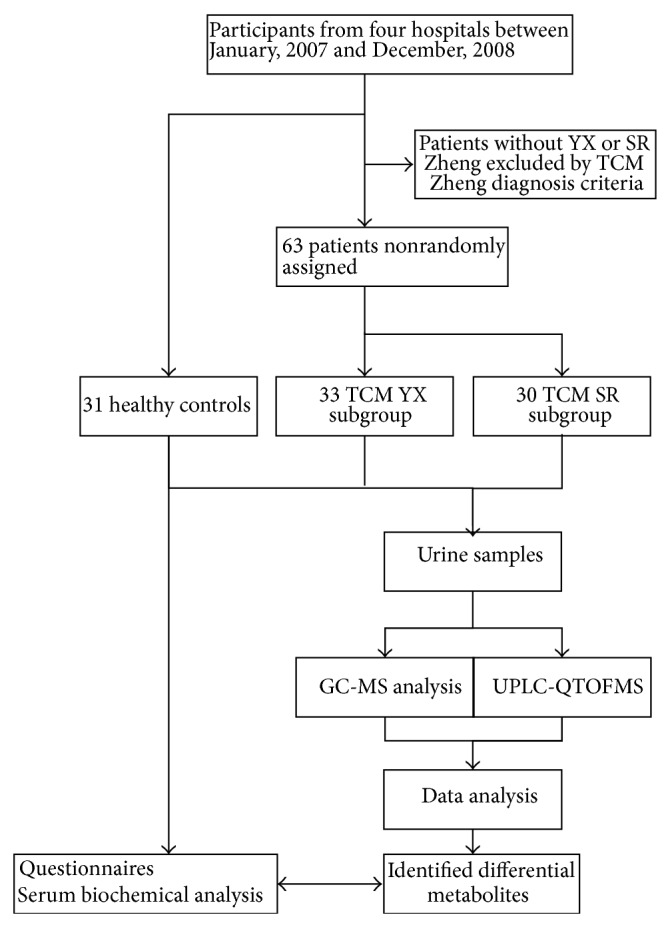
Scheme of the research design.

The interpretation for data analysis derived from GC-MS and UPLC-QTOFMS was performed by Laboratory of Systems Biomedicine (Ministry of Education), Shanghai Center for Systems Biomedicine, Shanghai Jiao Tong University, Shanghai, China. The verification of data was performed by Cancer Center, University of Hawaii, Honolulu, USA, which ensured that data were complete, accurate, and verifiable from source data. The trial profile is shown in Figure 1.

### 2.3. Statistical Analysis

For GC-MS, the resulting three-dimensional matrix data was imported to SIMCA-P 11.0 software (Umetrics, Umea, Sweden). Principle component analysis (PCA) was performed on the mean-centered and UV-scaled data to visualize general clustering, trends, and outliers among all samples on the scores plot. Partial least squares-discriminant analysis (PLS-DA) was used to maximize the variation. These differential metabolites selected from the PLS-DA model with VIP value (VIP > 1) are validated at a univariate level with Wilcoxon-Mann-Whitney test with a critical *P* value usually set to 0.05.

For UPLC-QTOFMS, the resulting three-dimensional matrix, including assigned peak index (retention time-*m*/*z* pairs), sample names (observations), and normalized peak area (variables), was then exported for multivariate statistical analysis using PLS-DA with the software SIMCA-P+ 12 (Umetrics, Umea, Sweden). Significant variables (markers) were selected based on a threshold of a multivariate statistical parameter (SIMCA-P 12.0 software, Umetrics, Umea, Sweden), such as VIP value (VIP > 1) from a typical 7-fold cross-validated PLS-DA model. These differential metabolites selected from the PLS-DA model were validated at a univariate level with Wilcoxon-Mann-Whitney test with a critical *P* value usually set to 0.05.

GraphPad Prism software (version 6.0) was used for data entry and management. All reported *P* values are two-sided, and a *P* value of less than 0.05 was considered significant. Data analyses were performed with SAS software (version 9.1).

## 3. Results

### 3.1. Clinical Characteristics of Liver Cirrhosis Patients

The clinical characteristics of cirrhosis patients including TCM YX group and SR group were summarized in Table 1. Serum levels of albumin (Alb) and apolipoprotein A-1 (APOA-1) in SR subgroup were lower than in YX subgroup. Serum level of total bilirubin (TBiL) in SR subgroup was higher than in YX subgroup. No statistical discrepancy was found in liver function between the two groups.

### 3.2. GC-MS Analysis

A total of 165 ion features were obtained and 11 identified urine metabolites were differentially expressed in cirrhotic patients compared to those in healthy controls. Peak intensity comparison of the differentially expressed metabolite levels in liver cirrhosis patients compared to those in healthy controls was summarized in Table 2.

**Figure 2:**
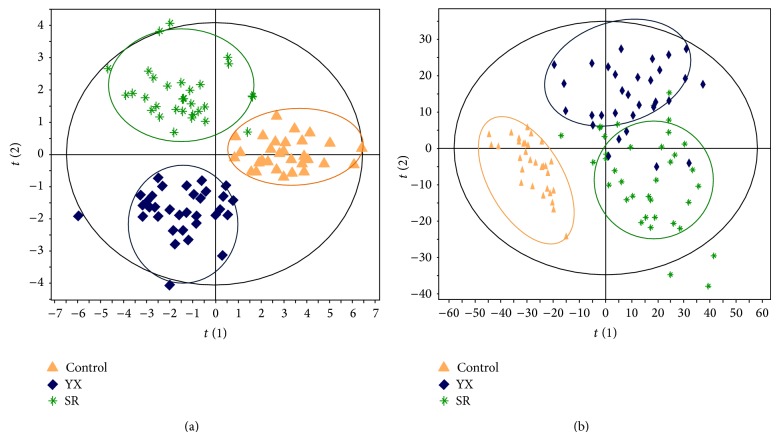
PLS-DA scores plot of urinary metabolites from healthy controls and TCM YX and SR Zheng patients with posthepatitis B cirrhosis using GC-MS spectral data (a) and UPLC-QTOFMS spectral data (b).

PCA and PLS-DA analysis were performed to distinguish healthy subjects from cirrhosis patients in TCM YX subgroup and SR subgroup. With the 165 features determined by GC-MS, a PCA scores plot (figure not shown) using 4 components (R2X = 0.486) and a cross-validated PLS-DA model using 1 predictive component and 2 orthogonal components (R2Xcum = 0.502, R2Ycum = 0.77, and Q2Ycum = 0.476) were constructed (Figure 2(a)). There appears to be separation between healthy controls and cirrhosis patients, reflecting the pathophysiological variations of liver cirrhosis disease. Moreover, the metabolite profiles of TCM YX or SR Zheng cirrhosis patients are also, respectively, different from the healthy controls, indicating the consistency between TCM Zheng and disease (Figure 2(a)). Compared to healthy controls, there were two urine metabolites, acetyl citrate, and 4-hydroxy-benzenepropanedioate, changed synchronously in two TCM Zheng subgroups of cirrhosis patients in all 11 identified differential expressed metabolites. Three metabolites, 2-pentendioate, citrate, and aconitate, appeared in TCM YX subgroup of liver cirrhosis patients, and two metabolites, 4-pyridinecarboxylate and hippurate, appeared in TCM SR subgroup (Table 2).

### 3.3. UPLC-QTOFMS Analysis

A PCA scores plot using 5 components (R2Xcum = 0.472, Q2cum = 0.058) and a cross-validated PLS-DA model using one predictive component and three orthogonal components (R2Xcum = 0.121, R2Ycum = 0.742, and Q2Ycum = 0.237) were constructed with 8,163 ion features detected on the UPLC-QTOFMS spectra. Clear separation among healthy controls, TCM YX subgroup, and SR subgroup of cirrhosis patients (Figure 2(b)) was obtained, suggesting that systemic metabolic variations in urine reflect the diversity between two TCM Zhengs.

A total of 28 characteristic urinary metabolites were identified from UPLC-QTOFMS negative ion mode for liver cirrhosis, as summarized in Table 2. Thereinto, higher peak intensity of two metabolites, L-aspartyl-4-phosphate and cortolone-3-glucuronide, were seen in TCM YX subgroup and one metabolite, glycoursodeoxycholate, was lower in SR subgroup compared to healthy controls (Table 2).

## 4. Discussion

Methods identifying the liver condition have been established in cirrhosis study, including the histological observations characterized by scar tissue, fibrous septa and regenerative nodules [39, 40], the clinical manifestation panels separating the compensated cirrhosis from the decompensated cirrhosis [41, 42], and CP scores classification reflecting the aggravated grade of the liver function [43]. All methods mentioned above aim to provide accurate diagnosis for disease and objective evaluation for the therapeutic schedule. With increasing requirement for the personalized treatment throughout the world, TCM Zheng stratification has become an important approach in disease diagnosis and treatment. Our previous study provided five TCM Zheng diagnostic criteria for the posthepatitis B cirrhosis according to clustering and merging of the clinical evidences [20], and, subsequently, patients with posthepatitis B cirrhosis were divided into five stratifications. Among the five TCM Zhengs, “Liver-Kidney Yin Deficiency” (YX) is a pure and representative “Deficiency Zheng,” while “Dampness-Heat Internal Smoldering” (SR) is a pure and representative “Excess Zheng.” “Deficiency” and “Excess,” the properties being entirely opposite, are the important guiding principles in TCM for analyzing the condition of the body's resistance to pathogenic factors. “Deficiency” refers to deficient healthy Qi and “Excess” refers to excessive pathogenic Qi. The other three TCM Zhengs are of combination status composed of “Deficiency Zheng” and “Excess Zheng,” although they differ in degree.

The liver is the most important metabolic organ in the human body, responsible for metabolism of a large array of substrates, such as sugar, protein, fat, and phytochemical compound [44]. The liver cirrhosis has been linked closely to metabolic disorders [45]. Metabonomics stays on the downstream terminal of system biology, contributing to an altered expression of a large number of metabolites at systemic level. Therefore, metabonomics is suitable for the study of liver disease. The present study is designed to characterize the alteration of urinary metabolite markers associated with TCM YX or SR Zheng in posthepatitis B cirrhosis and to provide the biological substance evidence for the TCM YX and SR Zheng stratification.

Two panels of markers, 11 and 28 urinary metabolites identified by GC-MS and UPLC-QTOFMS, respectively, were significantly altered in cirrhosis participants (Table 2). The PLS-DA models derived from our current GC-MS and UPLC-QTOFMS analysis showed good and similar separation between healthy controls and cirrhosis patients, or TCM YX and SR subgroups. The results in our study reflected the abnormal key metabolic pathways of energy metabolism, tricarboxylic acid (TCA) cycle, amino acid, bile acids, steroids, and intestinal microbial metabolism in the posthepatitis B cirrhosis patients. However, the abnormal alteration of metabolic pathways did not synchronously occur between TCM YX subgroup and SR subgroup patients. The abnormal alteration of aconitate, citrate, and 2-pentendioate was only found in TCM YX subgroup patients, but not in TCM SR subgroup. Aconitate is an intermediate in the tricarboxylic acid (TCA) cycle produced by the dehydration of citrate and the aconitase catalyses the stereospecific isomerization of citrate to isocitrate via cis-aconitate in the TCA cycle. The significantly elevated level of citrate and aconitate indicates an altered TCA cycle. TCA cycle is the final metabolic pathway of three major nutrients (protein, fat, and carbohydrates) to generate energy, whose changes represent the energy metabolism disorderly in the body. Thus, energy metabolism disorder may well be the biological cause of “soreness and flaccidity of waist and knees” in TCM YX Zheng and is more serious than that of TCM SR Zheng.

**Figure 3:**
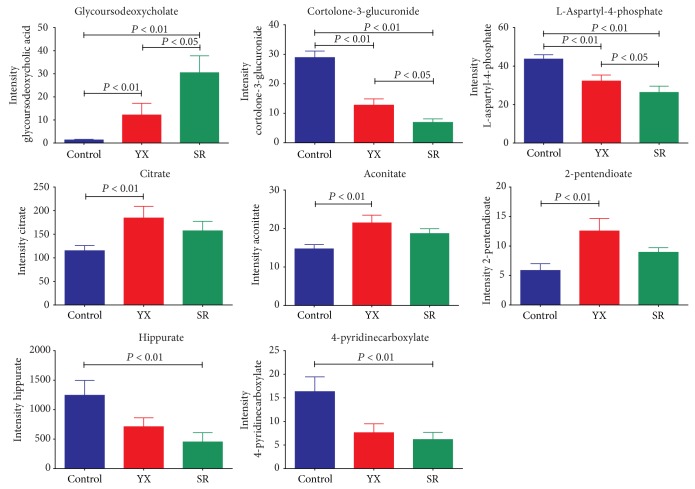
Bar charts of eight representative metabolite markers (mean ± SEM.) that are differentially expressed in healthy controls and TCM YX and SR Zheng patients with posthepatitis B cirrhosis.

On the contrary, the abnormal alteration of hippurate and 4-pyridinecarboxylate was only found in TCM SR subgroup patients, but not in TCM YX subgroup. Decreased hippurate in TCM SR subgroup patients and *α*-hydroxyhippurate in all cirrhotic subjects were generally produced via gut microbial-human cometabolism [46]. Change of hippurate showed that gut microbiota metabolism in TCM SR subgroup was damaged more significantly than that in TCM YX subgroup. Additionally, 3 urinary metabolites including glycoursodeoxycholate, cortolone-3-glucuronide, and L-aspartyl-4-phosphate (Figure 3) and 3 serum biochemical indices including Alb, TBiL, and APOA-1 (Table 1) were significantly altered between TCM YX subgroup and SR subgroup, suggesting that these metabolites and indices could be potential biomarkers for TCM YX and SR Zheng stratification of the posthepatitis-B cirrhosis patients. L-aspartyl-4-phosphate is a derivative from the interaction between L-aspartate and adenosine triphosphate (ATP). L-aspartate is usually used to improve resistance for fatigue in the body. Reduced levels of L-aspartyl-4-phosphate in cirrhotic patients should be associated with lower tolerance against fatigue. In clinic, the cirrhotic patients with TCM SR Zheng are indeed easier to feel fatigue than those with TCM YX Zheng. Serum TBiL levels have been an important jaundice evidence identifying TCM SR Zheng of many diseases, closely associated with ursodeoxycholic acid (UDCA) [47, 48] and intestinal flora [49]. Glycoursodeoxycholate is the secondary bile acid derived from UDCA conjugated with glycine. Elevated levels of glycoursodeoxycholate in urine of cirrhotic subjects indicated the reduced enterohepatic circulation and the lowered utilization rate of UDCA.

The liver's blood supply mainly comes from the intestine through the portal vein. The liver is vulnerable to exposure of bacterial products translocated from the gut lumen via the portal vein. Disruption of the intestinal barrier in developing liver cirrhosis results in the leaky gut, which contributes to bacterial translocation [50–52]. Increase in intestinal permeability leads to the translocation of intestine-derived bacterial products such as lipopolysaccharide (LPS) and unmethylated CpG containing DNA to the liver via the portal vein. Clinical evidence has demonstrated that elevated LPS level was found in the systemic and portal circulation in cirrhotic patients [53, 54]. Translocated LPS mediates Toll-like receptors (TLRs) activation in the liver, which are expressed on Kupffer cells, endothelial cells, dendritic cells, biliary epithelial cells, hepatic stellate cells, and hepatocytes. TLRs activate these cells to enhance liver inflammation and contribute to acute and chronic liver diseases. Alarmins, the products released from damaged cells or tissues, also trigger TLR signaling and cause inflammation without actual infections, referred to sterile inflammation [55]. Thus, the activation of TLR signaling through intestine-derived microbial products and alarmins may contribute to the progression of liver diseases [50]. And then, cirrhosis-mediated liver dysfunction may decrease the secretion of bile acids that causes bacterial overgrowth and may change bacterial composition in intestine [56, 57]. The analysis of fecal microbiome in patients with hepatitis B and alcoholic liver cirrhosis demonstrated an increase in pathogenic Enterobacteriaceae and Streptococcaceae, while beneficial bifidobacteria and Lachnospiraceae were decreased [58, 59]. For animals with cirrhosis, treatment with probiotics (e.g.,* Bifidobacterium*) reduces* Enterobacter*, while it increases* Bifidobacterium* and* Lactobacillus*, resulting in decreased systemic endotoxin levels and improved liver function [60]. In patients with chronic severe hepatitis, the concentration of plasma endotoxin positively correlated with levels of TNF-alpha, IL-1 beta, TBiL, and the number of fecal Enterobacteriaceae and negatively correlated with* Bifidobacterium* [61]. Our results further proved the correlation between intestinal flora, enterohepatic circulation, and liver damage in the posthepatitis B cirrhosis patients. The alteration of urinary metabolites, serum TBiL, Alb, and APOA-1 illustrated that the injury degree of systemic physiopathology in the cirrhotic subjects with TCM SR Zheng was worse than that with TCM YX Zheng. Cirrhosis with TCM SR Zheng could have a poor prognosis of the disease. Moreover, our study well supported the traditional pathogenesis theory of Chinese medicine, which with the accumulation of pathological products, Deficiency Zheng will transform into a preponderant condition of Excess Zheng in chronic diseases, if not treated or if therapy fails [62].

There are some limitations in our study. First, the included participants were restricted to men only because women are easier to suffer from hormone interference, such as menses and early pregnancy, and difficult to judge the pathological or physiological alteration of hormone associated with liver cirrhosis. Second, metabolites of great statistical significance warrant further validation and screening as potential biomarkers for posthepatitis B cirrhosis diagnosis and TCM Zheng stratification in larger group of participants with both genders and other TCM Zhengs.

## 5. Conclusions

In conclusion, our results suggest that a panel of unique urinary metabolite markers is of clinical potential for the disease diagnosis and patient stratification for liver cirrhosis. These metabolite markers reflect the essence of the patients with posthepatitis B cirrhosis characterized by the disorders of the TCA cycle, amino acid, bile acids, hormones, dopamine, intestinal microbial metabolism, and oxidative stress. Moreover, the energy metabolism disorder is special prominent in cirrhotic patients with TCM YX Zheng, while the abnormality of the dopamine, intestinal microbiota metabolism, and oxidative stress is more serious in those with TCM SR Zheng. Specific urinary metabolites may be used as the biomarkers for TCM Zheng stratification. One of the most remarkable things about this study is that metabonomic profiling, as a powerful approach, partly interprets the biological reasons inducing TCM Zheng manifestation. Cirrhosis with TCM SR Zheng manifests more serious changes in the physiopathology of disease. Enclosing the same cohort of participants, we have obtained more information by integrating two medicine systems, indicating that urinary metabolite variation not only is associated with the pathological progression of cirrhosis but also acted as evidence of TCM Zheng stratification, contributing to the personalized diagnosis or treatment.

## Figures and Tables

**Table 1:** Clinical information and characteristics of human subjects.

Variable	Control	Liver cirrhosis	TCM Zhengs of liver cirrhosis	
			YX subgroup	SR subgroup
Patients (*n*)	31	63	33	30
Age (y)	49.70 ± 5.46	52.95 ± 8.86	52.00 ± 8.82	53.83 ± 8.91
Body height (cm)	170.8 ± 3.05	171.5 ± 5.07	171.5 ± 5.40	171.5 ± 4.92
Body weight (kg)	67.48 ± 6.37	65.91 ± 9.26	67.31 ± 8.36	65.38 ± 9.521
BMI	23.14 ± 2.14	22.73 ± 2.78	22.83 ± 2.14	22.22 ± 3.020
RBC (10^12^/L)			3.60 ± 0.70	3.32 ± 0.68
WBC (10^9^/L)			4.44 ± 1.70	4.89 ± 2.74
HB (g/L)			119.5 ± 23.94	111.5 ± 19.40
NEUT# (10^9^/L)			2.41 ± 1.21	2.68 ± 1.01
LYM# (10^9^/L)			1.22 ± 0.72	1.31 ± 0.90
PLT (10^9^/L)			88.89 ± 45.84	85.09 ± 61.12
Alb (g/L)			32.62 ± 6.06	28.86 ± 6.18^**^
Glb (g/L)			35.10 ± 7.67	33.37 ± 9.0
A/G (100%)			0.98 ± 0.29	0.92 ± 0.29
ALT (IU/L)			71.73 ± 63.61	64.68 ± 52.57
AST (IU/L)			87.13 ± 67.05	79.58 ± 51.95
GGT (IU/L)			86.50 ± 75.19	73.35 ± 59.42
ALP (IU/L)			136.4 ± 94.59	110.5 ± 59.68
CHE (IU/L)			3345 ± 1296	3148 ± 1446
TBiL (*μ*mol/L)			42.92 ± 27.17	78.71 ± 37.11^**^
DBiL (*μ*mol/L)			15.59 ± 14.02	40.22 ± 35.16
PT (sec)			16.31 ± 1.99	17.41 ± 2.98
INR (%)			1.47 ± 0.24	1.62 ± 0.38
BUN (mmol/L)			5.80 ± 2.51	6.01 ± 2.91
Cr (*μ*mol/L)			83.37 ± 31.09	86.20 ± 33.49
TCH (mmol/L)			3.32 ± 0.84	3.27 ± 0.93
TG (mmol/L)			0.81 ± 0.23	0.90 ± 0.35
APOA-1 (g/L)			0.89 ± 0.22	0.76 ± 0.17^**^
FPG (mmol/L)			5.81 ± 1.91	5.69 ± 2.0
AFP (ng/mL)			49.60 ± 66.67	66.37 ± 132.2

Note: The results are presented as mean ± SD and were compared by *t*-test. ^**^ *P* < 0.01, TCM YX Zheng subgroup versus TCM SR Zheng subgroup.

BMI: body-mass index; RBC: red blood cell; WBC: white blood cell; HB: haemoglobin; NEUT#: absolute neutrophil Count; LYM#: absolute lymphocyte count; PLT: platelet; Alb: albumin; Glb: globulin; A/G: albumin/globulin; ALT: alanine aminotransferase; AST: aspartate aminotransferase; GGT: gamma glutamyl transferase; ALP: alkaline phosphatase; CHE: cholinesterase; TBiL: total bilirubin; DBiL: direct bilirubin; PT: prothrombin time; INR: international normalized ratio; BUN: blood urea nitrogen; Cr: creatinine; TCH: total cholesterol; TG: triglycerides; APOA-1: apolipoprotein A-1; FPG: fasting plasma glucose; AFP: alpha-fetoprotein.

**Table 2:** List of urinary differential metabolites in cirrhosis patients and among TCM YX, SR Zheng subgroup relative to controls.

Compounds	Liver cirrhosis versus control			YX versus control		SR versus control		SR versus YX	
	VIP^a^	FC^b^	*P* ^c^	FC^d^	*P* ^c^	FC^e^	*P* ^c^	FC^f^	*P* ^c^
GC-MS									
4-Pyridinecarboxylate	1.855	0.46	1.53*E* − 02	0.47	7.05*E* − 02	0.38	9.40*E* − 03	0.81	6.54*E* − 01
Threonine^*^	1.498	0.64	5.96*E* − 02	0.63	2.98*E* − 02	0.60	2.46*E* − 02	0.96	8.96*E* − 01
Proline^*^	1.474	1.30	1.79*E* − 01	1.57	2.94*E* − 02	1.79	3.50*E* − 03	1.14	3.97*E* − 01
Citrate^*^	1.33	1.50	8.28*E* − 03	1.60	1.39*E* − 02	1.37	1.39*E* − 01	0.85	3.33*E* − 01
Aconitate^*^	1.393	1.36	1.97*E* − 03	1.46	2.30*E* − 03	1.27	7.49*E* − 02	0.87	2.05*E* − 01
2-Pentendioate	1.734	1.58	1.95*E* − 02	2.14	2.00*E* − 03	1.52	1.57*E* − 01	0.71	9.28*E* − 02
Hippurate^*^	1.905	0.47	2.34*E* − 02	0.57	1.19*E* − 01	0.36	1.33*E* − 02	0.64	6.07*E* − 01
2-Aminobutyrate^*^	1.954	0.38	2.04*E* − 02	0.34	5.80*E* − 03	0.30	4.30*E* − 03	0.88	8.64*E* − 01
Acetyl citrate	1.517	2.75	2.44*E* − 03	3.26	9.30*E* − 03	3.24	1.15*E* − 02	1.00	9.87*E* − 01
3,4-Dihydroxyphenylacetate^*^	2.121	1.88	2.08*E* − 04	2.19	<0.0001	1.74	1.29*E* − 02	0.80	1.30*E* − 01
4-Hydroxy-benzenepropanedioate	1.723	4.29	6.84*E* − 04	4.41	1.65*E* − 02	5.51	2.20*E* − 03	1.25	4.40*E* − 01

UPLC-QTOF-MS									
cis-Aconitate^*^	2.0	0.75	6.30*E* − 05	0.74	<0.0001	0.76	<0.0001	1.03	6.99*E* − 01
Pyroglutamate^*^	2.1	0.69	7.74*E* − 07	0.65	<0.0001	0.75	<0.0001	1.15	1.02*E* − 01
O-Phosphotyrosine	2.0	0.70	3.71*E* − 06	0.72	<0.0001	0.70	<0.0001	0.97	7.59*E* − 01
3-Methoxy-4-hydroxyphenylglycol sulfate	2.1	1.70	3.15*E* − 09	1.73	<0.0001	1.72	<0.0001	0.99	9.40*E* − 01
Alpha-hydroxyisobutyrate^*^	2.4	0.42	1.28*E* − 06	0.46	<0.0001	0.35	<0.0001	0.75	2.13*E* − 01
3-Hydroxyisovalerate^*^	2.4	0.55	1.84*E* − 10	0.55	<0.0001	0.54	<0.0001	0.99	9.57*E* − 01
Dopaxanthin	1.8	0.23	7.15*E* − 04	0.30	2.00*E* − 04	0.14	<0.0001	0.46	3.84*E* − 01
Alpha-hydroxyhippurate^*^	2.1	0.35	8.93*E* − 06	0.42	<0.0001	0.24	<0.0001	0.57	1.38*E* − 01
Canavaninosuccinate	3.1	25.57	9.19*E* − 21	25.94	<0.0001	25.79	<0.0001	0.99	9.58*E* − 01
L-Aspartyl-4-phosphate	1.6	0.71	1.41*E* − 04	0.74	7.80*E* − 03	0.60	<0.0001	0.82	4.96*E* − 02
Isoxanthopterin	1.6	0.74	2.66*E* − 03	0.77	1.28*E* − 02	0.71	1.50*E* − 03	0.92	4.96*E* − 01
Tyrosine-betaxanthin	2.6	0.38	2.92*E* − 08	0.41	<0.0001	0.34	<0.0001	0.83	4.21*E* − 01
Estrone^*^	1.4	0.78	1.70*E* − 03	0.79	2.80*E* − 03	0.78	1.30*E* − 03	0.98	8.48*E* − 01
Glycocholic acid 3-glucuronide	1.7	5.18	7.02*E* − 07	5.37	5.00*E* − 04	5.30	5.00*E* − 04	0.99	9.52*E* − 01
Taurohyocholate^*^	1.5	119.52	2.29*E* − 07	119.94	2.90*E* − 03	150.74	2.00*E* − 04	1.26	4.20*E* − 01
Cortolone-3-glucuronide	2.5	0.36	2.32*E* − 09	0.44	<0.0001	0.24	<0.0001	0.54	2.95*E* − 02
Tetrahydroaldosterone-3-glucuronide	2.6	0.31	3.97*E* − 09	0.37	<0.0001	0.22	<0.0001	0.58	1.01*E* − 01
11-Beta-hydroxyandrosterone-3-glucuronide	2.4	0.38	2.13*E* − 07	0.44	<0.0001	0.31	<0.0001	0.70	1.76*E* − 01
N-Acetyl-leukotriene E4	2.6	0.12	2.82*E* − 06	0.13	<0.0001	0.08	<0.0001	0.60	6.79*E* − 01
11-Oxo-androsterone glucuronide	2.3	0.25	3.44*E* − 05	0.29	<0.0001	0.19	<0.0001	0.67	4.60*E* − 01
Glycocholate^*^	1.9	12.72	3.61*E* − 10	12.57	4.00*E* − 04	16.45	<0.0001	1.31	2.15*E* − 01
Dehydroepiandrosterone 3-glucuronide	2.4	0.29	1.45*E* − 07	0.33	<0.0001	0.20	<0.0001	0.60	2.32*E* − 01
Androsterone sulfate	2.5	0.01	1.28*E* − 05	0.00	<0.0001	0.02	<0.0001	234.20	9.11*E* − 01
Testosterone sulfate	2.3	0.21	4.09*E* − 05	0.21	<0.0001	0.19	<0.0001	0.90	8.74*E* − 01
Glycoursodeoxycholate^*^	1.3	16.41	1.30*E* − 04	9.19	8.00*E* − 03	23.03	2.00*E* − 04	2.51	1.57*E* − 02
Androsterone glucuronide	3.1	0.27	1.16*E* − 13	0.31	<0.0001	0.19	<0.0001	0.62	9.19*E* − 02
17-hydroxyandrostane-3-glucuronide	2.9	0.27	2.05*E* − 10	0.31	<0.0001	0.20	<0.0001	0.67	2.28*E* − 01
Glycolithocholate 3-sulfate	2.9	0.04	3.09*E* − 07	0.06	<0.0001	0.01	<0.0001	0.12	6.49*E* − 01

Note: ^*^Metabolites were verified by reference standards; ^a^variable importance in the projection (VIP) was obtained from PLS-DA model with a threshold of 1.0; ^b^fold change (FC) was obtained by comparing those metabolites in liver cirrhosis patients to controls; ^c^ *P* values were calculated from Wilcoxon-Mann-Whitney test; ^d^FC was obtained by comparing those metabolites in liver cirrhosis patients with TCM YX Zheng to controls; ^e^FC was obtained by comparing those metabolites in liver cirrhosis patients with TCM SR Zheng to controls; ^f^FC was obtained by comparing those metabolites in liver cirrhosis patients with TCM SR Zheng to TCM YX Zheng. FC with a value >1 indicates a relatively higher concentration present in liver cirrhosis patients or liver cirrhosis patients with TCM YX, SR Zheng while a value <1 means a relatively lower concentration as compared to the controls or TCM YX Zheng subgroup.
